# Magnetic Field Sensing Based on Whispering Gallery Mode with Nanostructured Magnetic Fluid-Infiltrated Photonic Crystal Fiber

**DOI:** 10.3390/nano12050862

**Published:** 2022-03-03

**Authors:** Chencheng Zhang, Shengli Pu, Zijian Hao, Boyu Wang, Min Yuan, Yuxiu Zhang

**Affiliations:** 1 College of Science, University of Shanghai for Science and Technology, Shanghai 200093, China; 211560115@st.usst.edu.cn (C.Z.); 201840111@st.usst.edu.cn (Z.H.); boyuwang2002@163.com (B.W.); ym19821237760@163.com (M.Y.); 192282050@st.usst.edu.cn (Y.Z.); 2 Shanghai Key Laboratory of Modern Optical System, University of Shanghai for Science and Technology, Shanghai 200093, China

**Keywords:** whispering gallery mode, microcavity resonator, magnetic field sensor, magnetic fluid, fiber sensor

## Abstract

A kind of novel and compact magnetic field sensor has been proposed and investigated experimentally. The proposed sensor consists of a tapered single mode fiber coupled with a nanostructured magnetic fluid-infiltrated photonic crystal fiber, which is easy to be fabricated. The response of magnetic fluid to magnetic field is used to measure the intensity of magnetic field via whispering gallery mode. The magnetic field-dependent shift in resonance wavelength is observed. The maximum magnetic field intensity sensitivity is 53 pm/mT. The sensor sensitivity is inversely proportional to the thickness of the photonic crystal fiber cladding.

## 1. Introduction

Compared with the traditional measurement methods, optical fiber magnetic field sensors have attracted great attention due to their outstanding virtues such as intrinsic safety, compact size, easy fabrication and immunity to electromagnetic interference. They have been widely applied in various fields such as electric power transmission, life science, medical health, geophysical research and military installations [1,2]. In principle, optical fiber is not sensitive to magnetic fields. Therefore, most of the proposed optical fiber magnetic field sensors realize magnetic field measurement by combining optical fiber with magnetic field sensitive materials [3].

Particularly, magnetic fluid (MF) is a kind of special nanostructured magneto-optic material, which consists of surfactant-coated magnetic nanoparticles and carrier liquid. It has unique optical properties such as dichroism, birefringence, Faraday effect and tunable refractive index under a magnetic field [4,5,6,7,8,9,10]. Furthermore, MF can be easily combined with optical fiber due to its fluidity. By infiltrating MF into the inner holes of microstructured fiber or coating the surface of specialty fiber with MF, many fiber-optic magnetic field sensors have been proposed based on various sensing technologies, such as multimode interference, Fabry–Perot interference, grating optics, Sagnac interference and surface plasma resonance [11,12,13,14]. The intensity of a magnetic field is usually measured by monitoring the wavelength shift of valley/peak in the output spectrum. Although these sensors have shown excellent sensing performance, the output spectral visibility is usually low and the full width at half maximum (FWHM) of the valley/peak is usually wide, which results in limited measurement accuracy and a low measurement resolution of magnetic field.

In recent years, the optical whispering gallery mode (WGM) resonator has shown enormous potential in sensing applications, such as biology [15,16,17], temperature [18,19,20], gas [21], humidity [22], laser [23], and magnetic field sensing [24,25,26,27,28,29,30,31,32,33,34]. Compared with the traditional optical resonators, the WGM resonator possesses high quality factor (Q), small mode volume and long lifetime of quantum photons. It is easy to achieve high-intensity output light with the WGM microcavity. Generally, the fabrication of WGM microcavity is based on mechanical deformation, while most of the mechanical processing would make fiber more fragile. Thus, the WGM tuning approach requires a delicate experimental apparatus. The tuning resolution is also rather limited. Those structures and methods may add the complexity of fabrication and increase the processing cost.

In this work, we propose and experimentally demonstrate a WGM sensor based on the photonic crystal fiber (PCF) infiltrated with MF. The MF serves as the magnetic field sensitive nanostructured material. The dependence of WGM resonance wavelength on magnetic field intensity is experimentally investigated. Furthermore, experimental results indicate that the influence of PCF diameter on the WGM resonant wavelength cannot be neglected. The proposed MF-infiltrated PCF WGM device possesses the features of high coupling efficiency and easy fabrication, which makes it a good candidate for application in magnetic field sensing and tunable optical filtering.

## 2. Fabrication and Experimental Details

**Figure 1:**
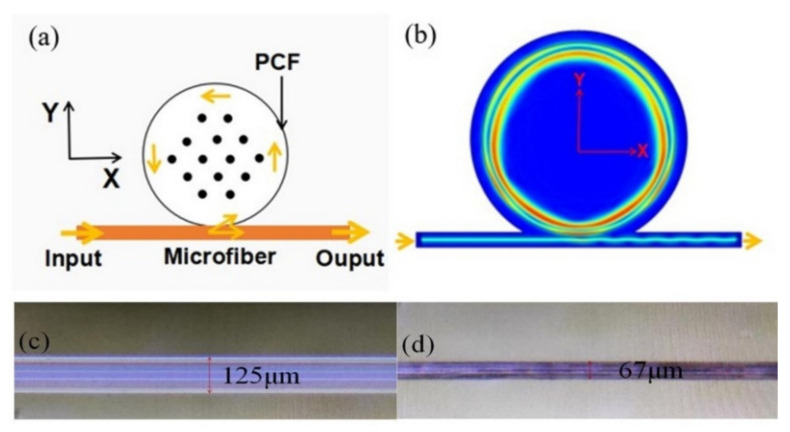
(**a**) Schematic diagram of the PCF infiltrated with nanostructured magnetic fluid coupled with a tapered microfiber. (**b**) Mode field distributions in x-y plane. (**c**) Micrograph of PCF before being infiltrated. (**d**) Micrograph of PCF after being infiltrated and corroded.

Figure 1a shows the schematic diagram of the PCF coupled with a microfiber, where the PCF provides the cylindrical microcavity. The microfiber is obtained by tapering a single mode fiber (SMF) with the heat-and-pull method [35]. The final diameter of the tapering region is 2 μm. Figure 1b shows the WGM field distribution in x-y plane. The WGMs are effectively excited by evanescent coupling between the microfiber and the PCF microcavity. The WGMs circulate along the inner equator of the microcavity.

Figure 1c shows the micrograph of unfilled PCF (core/cladding diameter is 7/125 µm). There are periodic arrangement of air holes existing in the cross-section of PCF (hole diameter is 3 µm). Infiltration of MF in the 1.5 cm-long section of PCF is carried out by dropping one end of the PCF into MF. The MF infiltrates the PCF air holes under the influence of capillary force. The fabrication process is carried out at ambient temperature and pressure condition. The employed MF is water-based MF with surfactant-coated 10-nm-diameter Fe_3_O_4_ nanoparticles (provided by Hangzhou Jikang New Materials Co., LTD, Hangzhou, China). The initial volume fraction is 33%. The refractive index (RI) of MF applied in this work is 1.36 and its volume fraction is around 9.7% (diluted with distilled water and measured by a refractometer (A670, Hanon, Jinan, China)). In theory, MF with higher concentration (viz. higher RI) may be greatly sensitive to the magnetic field. Then, two ends of the MF-infiltrated PCF are sealed with UV glue. The sealed PCF is put into the hydrofluoric (HF) acid solution with a concentration of 20%. The HF acid can corrode the outer wall of the sealed PCF. By controlling the corrosion time, cylindrical microcavity with uniform diameter could be obtained. Figure 1d shows the micrograph of the PCF after being infiltrated with MF and corroded by HF acid. The obtained PCF diameter (d) is about 67 μm. Experiments indicate that the HF begin permeating into the air holes and, hence, breaks the PCF microcavity when the corrosion time is longer than 70 min.

**Figure 2:**
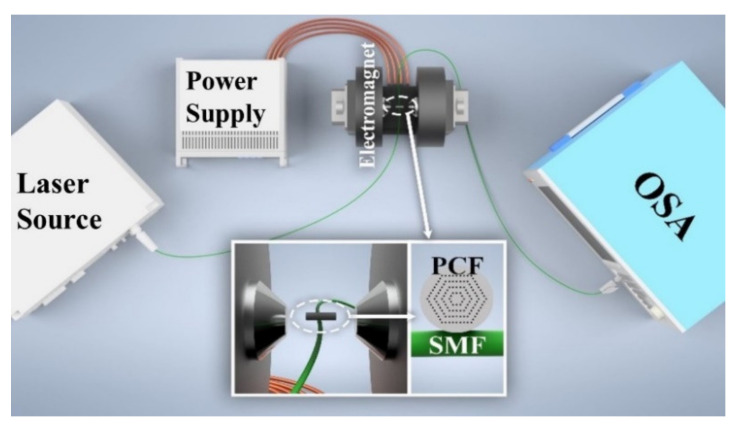
Schematic of experimental setup for investigating the sensing properties. SMF: single mode fiber, PCF: photonic crystal fiber, OSA: optical spectrum analyzer.

**Figure 3:**
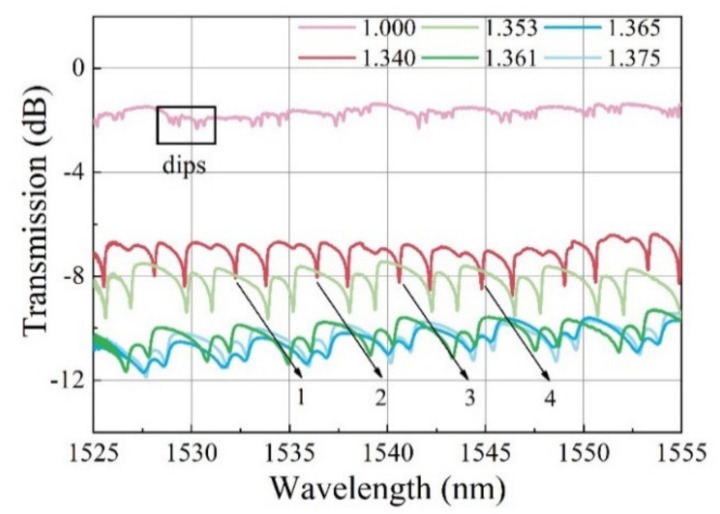
Transmission spectra of the sensing structure when the coupling region is immersed in glycerin solution of different refractive indices.

Figure 2 shows the schematic diagram of the experimental setup for investigating magnetic field sensing properties. An electromagnet and a highly stable light source with emitting light, covering wavelength range of 1450–1650 nm, were employed. The output power of the light source is 12 mW. The MF-infiltrated PCF WGM structure was placed in the middle of the electromagnet and carefully aligned to make the PCF parallel to the magnetic field. The magnetic field strength was adjusted by changing the magnitude of the supply current. It is measured simultaneously by a gauss meter with an accuracy of 0.1 mT. The transmission spectrum is monitored by the optical spectrum analyzer (OSA, Yokogawa AQ6370C, Tokyo, Japan). The dips near 1530 nm wavelength are selected for monitoring and analyzing (see the violet rectangle in Figure 3 below).

The coupling distance between PCF and microfiber is accurately controlled by two three-axis translation stages. The detailed process is as follows: first, one end of the SMF is connected with the light source and the other end is connected with the OSA. Then, two ends of the SMF tapering region are fixed on the glass slide with UV glue and the tapering region is partially suspended. Next, the PCF microcavity is fixed on the three-dimensional moving platform. Through adjusting the translation stage, the PCF microcavity can be moved slowly close to the tapering region for accurate coupling, which is synchronously observed by a CCD camera connected with the computer.

## 3. Sensing Principle and Experimental Results

The operating principle of the sensing structure is based on monitoring the shift of resonant wavelength. After the incident light enters the tapering region of the SMF, WGMs can be excited. WGM resonant wavelength should satisfy the following resonance condition [36].

(1) $$m\lambda \;=\;2\pi n_{eff}R$$

where *m* is an integer, *λ* is the resonant wavelength, *n_eff_* refers to effective refractive index (ERI) and *R* represents the outer radius of the PCF microcavity. Under the condition of fixed microcavity radius, resonant wavelength *λ* is affected by ERI *n_eff_*. The relationship between *λ* and *n_eff_* is given as

(2) $$\Delta \lambda =\left(\frac{\lambda }{n_{eff}}\right)\Delta n_{eff}$$

Equation (2) indicates that the resonant wavelength will shift towards the long wavelength with the increase in ERI.

To verify the above theory, the influence of ERI on resonant wavelength has been experimentally investigated and the results are shown in Figure 3. The sensing device consists of a tapered microfiber and a MF-infiltrated PCF microcavity (1.5 cm long, 125 μm in diameter) was placed on the experimental platform. The ambient RI near the coupling region was changed by dropping glycerin solution with different concentrations. When the ambient RI increased from 1.00 to 1.375, the resonant wavelength shifted towards the long wavelength direction (see the black arrows labeled as 1, 2, 3, 4 in Figure 3). The experimental results are in good agreement with the theory, which indicates that the sensor is sensitive to the change in the ambient RI.

**Figure 4:**
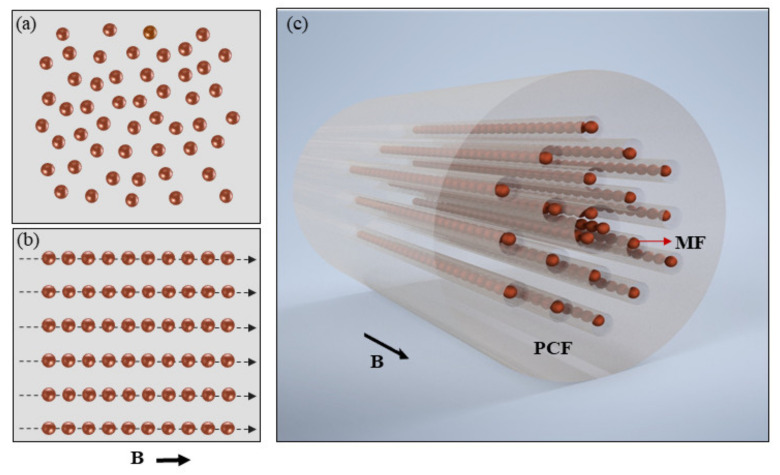
(**a**) Schematic of magnetic nanoparticles within magnetic fluid under zero magnetic field. (**b**) Schematic of magnetic nanoparticle columns within magnetic fluid under applied magnetic field. (**c**) Magnetic nanoparticle column distribution in PCF.

For magnetic field measurement, MF is employed as the magnetic field sensitive material. MF has both the magnetic properties of solid magnetic materials and the fluidity of liquid materials. Under zero magnetic field, the magnetic nanoparticles within MF are dispersed randomly and uniformly in carrier liquid, as shown in Figure 4a, which are assigned to the influence of surfactant and Brownian motion of magnetic nanoparticles. When the magnetic field is applied, the magnetic nanoparticles within MF are rapidly magnetized. Due to the dipolar interaction of inter-nanoparticles, the magnetic nanoparticles favor agglomeration along the magnetic field direction and form nanochain-like structures [37,38,39]. Figure 4b schematically shows the chains along the field direction. Figure 4c shows the magnetic nanoparticle chain distribution in PCF under applied magnetic field. Then, the dielectric constant and hence the RI of MF will change with magnetic field. The relationship between RI of MF and magnetic field is given by the following Langevin-like function [40]

(3) $$n_{MF}(H)=\left\{\begin{array}{cc} n_{0}, & H<H_{c,n}\\ (n_{S}-n_{0})\left[\coth(\alpha \frac{H-H_{c,n}}{T})-\frac{T}{\alpha (H-H_{c,n})}\right] & +n_{0},H>H_{c,n} \end{array}\right.$$

where *n_0_* is the RI of MF at zero magnetic field. *n_s_* is the saturated value of MF’s RI. *H* is the magnetic field strength in Gs. *H_c,n_* is the critical value of magnetic field strength. *T* is the thermodynamic temperature in Kelvin. *α* is the adjusting parameter. Thus, the resonant wavelength will shift with the magnetic field, which is fundamental for magnetic field sensing.

**Figure 5:**
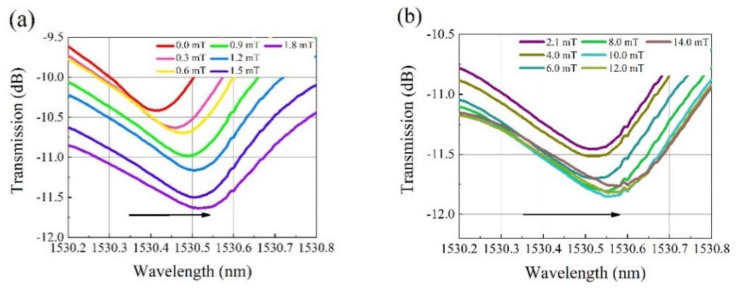
Transmission spectra at different magnetic field intensities for the PCF microcavity with diameter of 67 μm. The magnetic field strength is in the range of (**a**) 0 to 1.8 mT and (**b**) 2.1 to 14 mT.

**Figure 6:**
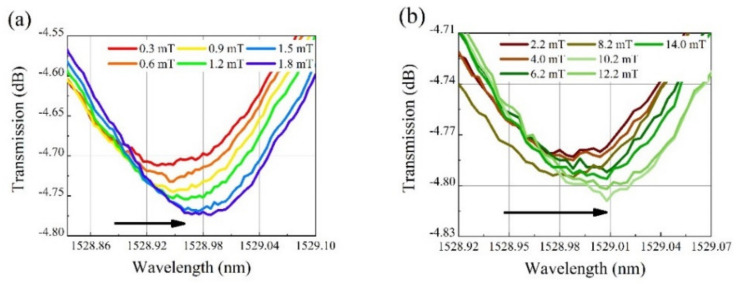
Transmission spectra at different magnetic field intensities for the PCF microcavity with diameter of 75 μm. The magnetic field strength is in the range of (**a**) 0.3 to 1.8 mT and (**b**) 2.2 to 14 mT.

Figure 5 and Figure 6 show the transmission spectra of the as-fabricated sensing structures under different magnetic field intensities. The diameters of the PCF microcavity are 67 and 75 μm, respectively. When the magnetic field intensity changes from 0 to 1.8 mT, the resonant wavelength changes greatly, as shown in Figure 5a and Figure 6a. Then, as the magnetic field intensity further increases to 14 mT, the resonant wavelength redshifts further as shown in Figure 5b and Figure 6b, but the shift speed decreases remarkably.

**Figure 7:**
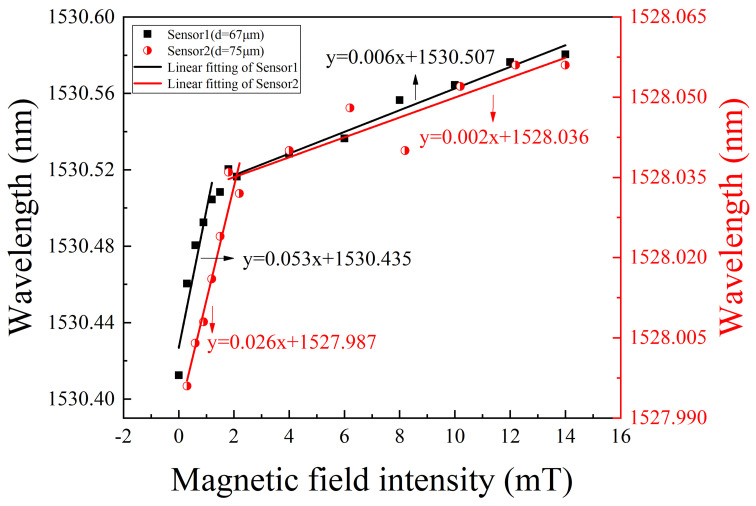
WGM resonant wavelength as a function of applied magnetic field for the PCF microcavity with diameter of d = 67 and 75 μm, respectively.

Figure 7 explicitly shows the corresponding resonant wavelength as a function of magnetic field intensity. Figure 7 indicates that, in the magnetic field range from 0 to 1.8 mT, the linear responsivities of the two sensing structures are 53 pm/mT and 26 pm/mT, respectively. However, in the magnetic field range from 2 to 14 mT, their linear responsivities are 6 pm/mT and 2 pm/mT, respectively. The maximum sensitivity is obtained to be 53 pm/mT. Higher sensitivity is obtained at weak magnetic field. At high magnetic field, the sensitivity decreases, which may be assigned to the tendency to saturation state of MF. In addition, the sensitivity is inversely proportional to PCF microcavity diameter. The smaller the PCF microcavity diameter is, the higher the sensitivity will be. Therefore, the proposed WGM sensors with higher sensitivity can be obtained by reducing the cladding thickness of the PCF microcavity.

For comparison, Table 1 lists the sensing structures, fabrication methods and sensing performance of the related optical fiber WGM magnetic field sensors. The mainly adopted structures and fabrication methods include pressure-assisted arc discharging on silica capillary or hollow fiber to obtain microbottles, or adjusting related fusion parameters on SMF to fabricate microspheres. It is obvious from Table 1 that the sensitivity of the proposed PCF WGM sensor is better than those of the structures based on hollow microbubble, borosilicate glass, microsphere and hollow microbottle, but lower than those of the structures based on silica microcapillary, fuse-discharging silica capillary and side-polished SiO_2_ microsphere. However, compared with the above-mentioned structures and fabrication methods, the PCF microcavity employed in this work has natural cylindrical microcavity and the fabrication process is simple and easy to repeat. Thus, the MF-infiltrated PCF WGM sensor is promising for highly sensitive weak magnetic field measurement in the future.

## 4. Conclusions

In summary, a kind of magnetic field sensor based on WGM excited in a MF-infiltrated PCF resonator has been demonstrated. The maximum magnetic field intensity sensitivity is obtained to be 53 pm/mT. Experimental results indicate that the cladding thickness of PCF microcavity influences the sensitivity of the sensor. The proposed device possesses the desirable features of high coupling efficiency and easy fabrication, which make it promising for application in magnetic field sensing and tunable optical filtering. The PCF-based WGM resonance structure is also simple and compact.

## Figures and Tables

**Table 1:** Sensing performance of various optical fiber WGM magnetic field sensors.

Sensing Structure	Fabrication Method	Maximum Sensitivity	Ref.
Hollow microbubble	Fuse-and-blow technique	0.081 pm/mT	[24]
Borosilicate glass	HF etching + silicon nitride ceramic heating + glassblowing method	4.0 pm/mT	[25]
Microsphere	HF etching + fusing + electrode discharging method	5.036 pm/mT	[26]
Silica microbubble	Fusing + discharging method	15.1 pm/mT	[27]
Hollow microbottle	Pressure-assisted air discharging + HF etching method	25.21 pm/mT	[28]
PCF	Tapering method	−61.86 pm/mT	[29]
Silica microcapillary	Hydrogen flame heating + air-pumped swelling method	75.7 pm/mT	[30]
Silica capillary	Fuse-discharging + increasing gas pressure + heated and soften method	8.45 pm/Gs (84.5 pm/mT)	[31]
SiO_2_ microsphere	Side-polishing method	0.0108 nm/Oe (108 pm/mT)	[32]
PCF	HF etching method	53 pm/mT	This work

## Data Availability

The original contributions presented in the study are included in the article. Further inquiries can be directed to the corresponding author.
